# Comprehensive Expression Profiling Analysis of Pituitary Indicates that circRNA Participates in the Regulation of Sheep Estrus

**DOI:** 10.3390/genes10020090

**Published:** 2019-01-28

**Authors:** Xiaoyue Li, Cunyuan Li, Junchang Wei, Wei Ni, Yueren Xu, Rui Yao, Mengdan Zhang, Huixiang Li, Li Liu, Hanli Dang, Wureli Hazi, Shengwei Hu

**Affiliations:** 1 College of Life Sciences, Shihezi University, Shihezi 832003, Xinjiang, China; 18799297836@163.com (X.L.); 18738595903@163.com (C.L.); 18899592515@163.com (J.W.); 15136705752@163.com (Y.X.); 15739330255@163.com (R.Y.); mdzzhang@163.com (M.Z.); 15739333350@163.com (H.L.); 15609936261@163.com (L.L.); 13199931067@163.com (H.D.); 2 College of Animal Science and Technology, Shihezi University, Shihezi 832003, Xinjiang, China; shzvhz@126.com

**Keywords:** sheep, circRNAs, estrus, anestrus, anterior pituitary

## Abstract

The pituitary gland is the most important endocrine organ that mainly regulates animal estrus by controlling the hormones synthesis. There is a significant difference between the estrus state and anestrus state of sheep pituitary system. Here, we studied the circular RNA (circRNA) expression profiles of the anterior pituitary of estrus and anestrus sheep using RNA-seq technology. Through this study, we identified a total of 12,468 circRNAs and 9231 differentially expressed circRNAs in the estrus and anestrus pituitary system of sheep. We analyzed some differentially expressed circRNAs by reverse transcription quantitative-PCR (RT-qPCR), and some circRNAs were demonstrated using RNase-R+ resistance experiments. CircRNAs involving the regulation of estrus-related terms and pathways are enriched by using gene ontology (GO) and Kyoto Encyclopedia of Genes and Genomes (KEGG) enrichment analysis. In addition, we also predicted partial microRNA-circRNA interaction network for circRNAs that regulate sheep estrus. Overall, this study explored a potential substantial role played by circRNAs involved in pituitary regulation on sheep estrus and proposed new questions for further study.

## 1. Introduction

Most sheep are seasonal estrus animals, and the reproductive rate of sheep is closely related to the time and frequency of estrus [1]. Previous studies have shown that there are significant physiological and endocrinological differences in the pituitary, when comparing estrous cycling ewes with anestrus ewes [2]. It is well known that almost all reproductive hormones are secreted by the hypothalamus-pituitary-gonad axis, such like gonadotropin-releasing hormone (GnRH), follicle-stimulating hormone (FSH), luteinizing hormone (LH), growth hormone (GH) [3,4]. Interestingly, references have shown that these reproductive-related hormones’ secretion levels are different between the estrus and anestrus periods of the ewes. For example, Zhang et al. showed that the levels of hormones such as progesterone, estrogen, LH and FSH were higher in the estrus phase of Kazakh sheep [5]. Most of these hormones are proteins and are primarily regulated in the synthesis and secretion at the transcriptional and post-transcriptional levels, which leads us to take into account the relevant non-coding RNAs involved in this process. Non-coding RNAs, including a variety of microRNAs (miRNAs), long non-coding RNAs (lncRNAs) and circular RNAs (circRNAs), are widely involved in post-transcriptional gene expression regulation and various biological processes [6,7]. CircRNA is also an important member of non-coding RNAs and may be an important molecule regulating gene expression, but circRNAs have rarely been studied in the past decades, especially in animal breeding, although microRNAs and lncRNAs have been widely studied as functional molecule regulating gene expression for many years [8,9]. In addition, as a new class of small RNA molecules in the eukaryotic transcriptome, circRNAs have stable properties and a closed-loop structure [10,11]. Interestingly, most circRNAs are composed of multiple exons and are conserved among different species, while expressing spatial and temporal specificity at different tissue and developmental stages of the same species. Recent studies have shown that circRNAs may function in the following modes: as an endogenous competitive RNA to regulate free miRNAs; using its own internal ribosome entry site (IRES) to translate and encode proteins [12,13].

Although extensive studies have been conducted on the regulation of GH transcription in the pituitary in the past few decades, the role of circRNAs in this regulation process remains unclear [14]. Previous studies have revealed multiple genes and pathways involved in the regulation of pituitary-associated genes, but there is no evidence that circRNA in the pituitary is involved in the GH regulation process [15,16]. Therefore, we hypothesized that there are differentially expressed circRNAs between the estrus state and anestrus state of sheep pituitary, which will provide a basis for understanding the role of circRNA in the pituitary in regulating these two physiological states. In order to fully explore and understand the role of circRNAs in pituitary regulation, we determined circRNA expression profiles in estrus and anestrus in sheep. In this study, we investigated the differential expression of circRNAs in the pituitary gland in estrus and anestrus in order to provide new insights into understanding the transition between estrus and anestrus in sheep from the perspective of circRNAs [3,5,17,18].

## 2. Materials and Methods

### 2.1. Ethics Statement

All experimental operations for the sheep involved in this study were performed in accordance with the Regulations for the Management of Laboratory Animals (Chinese State Council No. 676; revised in March 2017) and were carried out with approval from the Animal Protection and Use Committee of Shihezi University (SU-ACUC-08032).

### 2.2. Animals

Kazakh sheep are seasonal estrus animals, and estrus usually occurs in the fall, extending from September to February, and the rest of the time is anestrus [19]. The complete estrus cycle lasts approximately 18 days [20,21]. The average estrus interval for sheep is 16.5–17.5 days [19]. Healthy Kazakh ewes were selected to be placed in the natural pastures of Shihezi, Xinjiang, China. In the estrus season (September, Xinjiang, China), three estrus ewes (about 1.5 years old and weighing about 45 kg) were selected. In the anestrus season (April, Xinjiang, China), three anestrus ewes (about 1.5 years old and weighing about 45 kg) were selected. Beginning one month before the estrus period, the rams and ewes are placed together at least 15 min a day, and estrus is monitored twice daily according to the obvious estrus sign [22,23,24]. The ewes are visually measured by experienced technicians. When the ewes showed strong standing reflexes (without any movement) after the back pressure test, the vulva became red and the external genitalia protruded, accompanied by a yellow transparent viscous liquid discharged from the vulva, which was defined as estrus [22]. During our first observation of estrus (the first day of the estrus cycle), three ewes were selected for slaughter for anterior pituitary collection. When there is still no sign of estrus for more than 36 days (the time of two estrus cycles), it is defined as a period of anestrus [21]. During this time, three anestrus ewes were slaughtered for anterior pituitary collection. All pituitary samples were frozen in liquid nitrogen immediately after removal in a sterile environment and stored at −80 °C until total RNA was extracted.

### 2.3. RNA Extraction and Quality Test

According to the manufacturer’s instructions, estrus and anestrus sheep pituitary tissues were homogenized separately in liquid nitrogen, and total RNA was extracted using Trizol (Invitrogen, Carlsbad, CA, USA). The amount and purity of total RNAs were ensured by the Bioanalyzer 2100 system and the RNA 6000 Nano kit (Agilent Technologies, Santa Clara, CA, USA). The rRNA in total RNAs was removed prior to sequencing using an Epicentre Ribo-zero rRNA removal kit (Epicentre, Madison, WI, USA). The rRNA-depleted RNAs were digested with Rnase-R (RNR-07250, Epicentre) to remove linear RNAs, and the remainder were used to synthesize double-stranded cDNA using cDNA synthesis kit (Takara, Dalian, China). The cDNAs were end-repaired and ligated to the sequencing adaptor, and the desired fragments were selected. Subsequently, a PCR was performed to enrich the appropriate single strand to establish a library, then quality testing was performed by the Agilent Bioanalyzer 2100 (Agilent) system. Finally, all eligible cDNA libraries for pituitary tissues were sequenced using Illumina HiSeq PE2500 platform.

### 2.4. Circular RNAs Identification

In order to ensure the quality of data analysis, all the data of the machine underwent strict quality control. Clean data were obtained by removing the adapter sequence contaminants and low quality readings (including readings containing more than 10% of unknown bases, readings containing adaptors, and readings containing more than 50% of low-mass bases (with a Phred score of <5%) from the raw data. Use TopHat v2.0.9 (http://tophat.cbcb.umd.edu/) to align the data with the sheep reference sequence for the fastq file of the clean data, and the reference genome and gene annotations were downloaded from the sheep reference genome within the UCSC Genome Browser (http:/genome.ucsc.edu/). To identify circRNA, a 20-nt anchor sequence was selected from both ends of the reads that were never aligned with the reference sequence, respectively, and each pair of anchor sequences was again matched to the reference sequence using FindCirc software [14]. The reads with the 5′ end (start site and termination site were marked A3 and A4, respectively) of the anchor sequence were reverse matched to the downstream of the site, while the 3′ end (start site and termination site were marked A1 and A2, respectively) matches upstream of the site, while the splice site GT/AG is contained between A2 and A3, which was considered candidate circRNAs. Finally, those of the candidate circRNAs with reverse splicing reading frame greater than or equal to 2 were identified as true circRNAs.

### 2.5. Analysis of Differential Expression Circular RNAs

The expression amount of identified circRNAs was analyzed using TPM (transcripts per million; circRNAs were normalized by TPM) [25]. TPM density distribution analysis was performed to comprehensively examine the gene expression patterns of these circRNAs, and difference analysis of circRNA expression levels using DEGseq [26]. In order to screen out differentially expressed circRNAs, and considering the false positive rate, the threshold was set as q-value < 0.01 and |log2 (foldchange)|> 1 by default.

Furthermore, the amount of circRNAs expression in both states were analyzed by cluster analysis to estimate the clustering pattern of the differentially expressed circRNAs. Chromatographic cluster analysis was then used to show the combined expression level distribution of all differentially expressed circRNA groups due to TPM values for each group. In addition, statistical analysis was performed to estimate the number of differentially expressed circRNAs in each group. 

### 2.6. Gene Ontology, Kyoto Encyclopedia of Genes and Genomes Enrichment Analysis and Binding Sites Prediction

Kyoto Encyclopedia of Genes and Genomes (KEGG) enrichment analysis and GO (gene ontology) analysis websites were used to perform enrichment analysis of differentially expressed circRNAs of two states, and the host genes of differentially expressed circRNAs from each group were performed for enrichment analysis using GO and KEGG enrichment, respectively, according to the correspondence between circRNA and its host gene. In order to obtain more accurate results, the method GOseq was selected to perform GO enrichment analysis. With regard to KEGG, significance enrichment analysis of pathway was used to confirm both the most essential biochemical metabolic pathway and signal transduction pathway. Scores with *p* < 0.05 were considered significant for enrichment analysis.

CircRNA can inhibit functional miRNA by binding to miRNA [27]. To better understand the mechanism of microRNA binding to circRNA, we predicted miRNA-circRNA interaction networks using miRanda software (http://www.microrna.org/miranda_new.html). The results were exhibited by cytoscape 3.5.1 (http://www.cytoscape.org/).

### 2.7. Reverse Transcription PCR Analysis, RNase-R Treatment and Quantitative PCR Analysis

Total RNAs were extracted from pituitary of sheep using TRIzol (Invitrogen). From purified RNA, cDNA was synthesized using reverse transcription PCR (RT-PCR) Kit (Takara). RT-PCR was conducted using specific primers for 10 circRNAs (including circ_4723, circ_7336, circ_3004, circ_4460, circ_7204, circ_1993, circ_0198, circ_1655, circ_3088 and circ_3479). Amplification products of RT-PCR were analyzed by electrophoresis and DNA sequencing to confirm the presence of circRNAs. Total RNA was treated with RNase-R^+^ (RNR-07250, epicenter) prior to cDNA synthesis to detect resistance of circRNA to RNase-R digestion. Quantitative PCR (RT-qPCR) was used to verify differential expression. All identified circRNAs were subjected to qRT-PCR analysis using SYBR Green (Takara) according to the manufacturer’s protocol. We choose linear β-actin as an internal reference to normalize the expression of circRNAs. Three independent experiments were performed on triplicate samples. All primer sequences are shown in Table S1. PCR was conducted using the following reaction system: 10 μL of the premix (Takara), 2 μL of the cDNA template, 0.6 μL of the upstream and downstream primers, respectively, and 6.8 μL of RNase-free ddH_2_O. PCR was performed using the following thermocycling conditions: an initial denaturation at 95 °C for 3 min, followed by 45 cycles at 95 °C for 10 s, 58 °C for 15 s, and 72 °C for 5 s.

## 3. Results

### 3.1. Sequencing Quality Description

We obtained circRNAs expression data of Kazakh sheep pituitary in both estrus (PGN) and anestrus (PGA) states by RNA-seq. More than 210 million raw reads were detected in the pituitary glands of estrus and anestrus sheep. Since the quality of RNA-seq data directly affects subsequent bioinformatics analysis, we examined and estimated the quality of the data. Through quality control, we can see that the error rate of PGA and PGN are generally less than 0.5%, except for the front end of the sequence reading (mainly because the random primer base does not completely bind to the RNA template, or because of the reason the sequencer itself is inevitable under current conditions) (Figure S1). Statistics on the reads of GC content showed that the average values of PGN and PGA were 51.95% and 51.60%, respectively. Finally, clean data of more than 30.26 GB were obtained by removing the adapter reads and low quality reads, and these clean reads were used for the next bioinformatics analysis.

### 3.2. Screening and Identification of Circular RNAs

**Figure 1:**
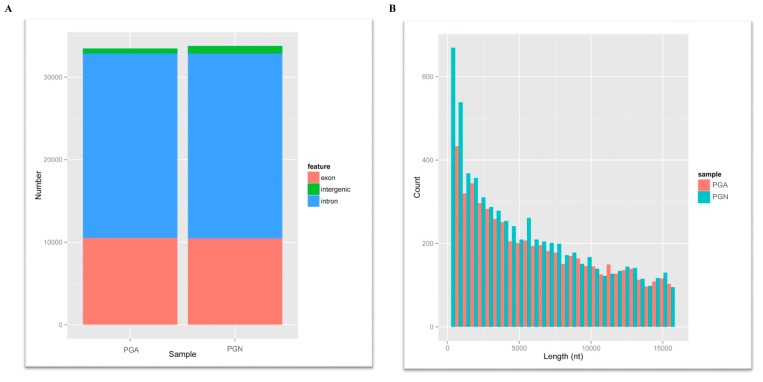
The information of circRNAs. (**A**) Length distribution of circRNAs for all samples. The abscissa is the length of the circRNA, and the ordinate indicates the abundance of the circRNA. Red for anestrus (PGA) and green for estrus (PGN). (**B**) Statistical graph of circRNA origin for all samples. Red stands for exons, blue stands for introns and green stands for intergenic. circRNAs: circular RNAs.

To understand the characteristics, abundance and differences of circRNAs in the pituitary gland during estrus and anestrus in sheep, we used the FindCirc (https://omictools.com/find-circ-tool) calculation pipeline to detect circRNAs using clean data after strict quality control, and the candidate circRNAs with a reads greater than 2 were identified as circRNA [14]. From the RNAseq of the PGA and the PGN, we identified a total of 12,468 circRNAs from these data, and the length of these circRNAs varies from ten to thousands of bases, but mainly concentrates below 10,000 bases (Figure 1A). In addition, we studied the composition of these circRNAs. As a result, the percentage of introns, intergenic regions, and exons that constitute circRNA in PGA were 30.9%, 66.4%, and 2.7%, respectively, while the percentages in PGN were 31.4%, 66.8%, and 1.8%, respectively (Figure 1B). However, that reveals no significant difference in the composition of circRNAs between PGA and PGN but shows a minimal percentage of exons in both states. Analysis of these circRNA sources revealed that these circRNAs were distributed on 26 autosomes and X chromosomes. All circRNAs annotations, chromosomal location, and host mRNAs are shown in Table S2.

### 3.3. Validation of Sheep Circular RNAs

**Figure 2:**
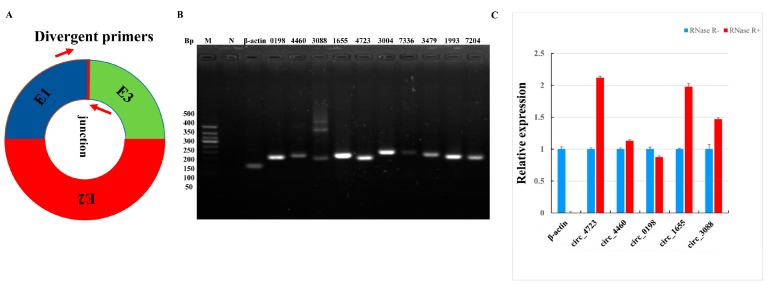
Validation of circRNAs between PGA and PGN. (**A**) Divergent primers used in the amplification of circular junctions. Red arrows represent divergent primers. (**B**) Electropherogram of reverse transcription polymerase chain reaction (RT-PCR) amplification of circRNAs with divergent primers. M is a marker (Tangene 50bp: 500 bp, 400 bp, 350 bp, 300bp, 250bp, 200 bp, 150 bp, 100 bp and 50 bp), and N is a negative control. (**C**) RNase R digestion resistance test of circRNA by quantitative PCR (RT-qPCR). β-actin was used as a linear control. The error bars represent ±SD.

Although we have obtained a large number of circRNAs in the estrus and anestrus pituitary gland of sheep through bioinformatics analysis, these analyses must be confirmed by some further experiments. First, in order to validate these circRNAs, we designed some divergent primers for their junction sites to prove the existence of these circRNAs (Figure 2A). Analysis of the results of RT-PCR amplification of 10 randomly selected circRNAs by agarose electrophoresis revealed that a single band of the expected size was amplified (Figure 2B). Some reactions generated non-specific products, which could be isoforms of alternative splicing circularization [14]. Next, we performed DNA sequencing on these amplified products, and the DNAMAN software (Version 5.2.2; Lynnon BioSoft, San Ramon, CA, USA) was used to compare the sequencing results with the head-to-tail linkage of these circRNAs to further confirm the existence of these circRNAs. In addition, we performed RNase-R^+^ digestion on five randomly selected circRNAs and tested the resistance of circRNAs to RNase-R^+^ digestion by RT-qPCR. The results indicate that compared to linear β-actin these five tested circRNAs all showed varying degrees of resistance to RNase-R^+^ digestion (Figure 2C). These results further confirm the accuracy of our informatics analysis.

### 3.4. Analysis and Validation of Differentially Expressed Circular RNAs between Anestrus and Estrus

**Figure 3:**
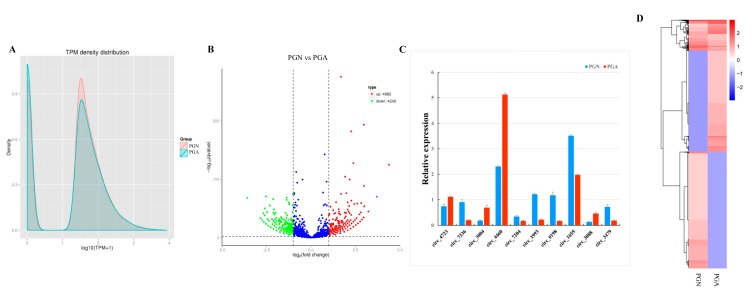
Analysis and validation of differentially expressed circRNAs between PGA and PGN. (**A**) CircRNAs expression amount transcript per million (TPM) density distribution map. The abscissa is the log10 (TPM+1) value of the circRNA, and the ordinate is the density corresponding to log10 (TPM+1). (**B**) Volcanic maps of differentially expressed circRNAs. The abscissa represents the fold change in expression of circRNAs in PGN and PGA, and the ordinate represents the statistically significant degree of change in the amount of expression of circRNAs. Blue dots indicate no significant differences in circRNAs, red dots indicate significantly up-regulated differences in circRNAs, and green dots indicate significant down-regulated differences in circRNAs. (**C**) Expression of 10 differentially expressed genes by RT-qPCR (*p* < 0.05). (**D**) Hierarchical clustering analysis of differentially expressed circRNAs. Red indicates high expression of circRNAs, blue indicates low expression of circRNAs.

We computed total reads that mapped to reference sequence along with their percentage in clean reads, including the multiply mapped reads, and uniquely mapped reads. The expression amounts of identified circRNAs were analyzed using TPM. In order to initially examine the gene expression pattern of the sample from the overall level, we performed a general analysis of the two groups of samples using the TPM density distribution (Figure 3A). Further analysis identified 9231 circRNAs differentially expressed between the estrus (PGN) and the anestrus (PGA) in sheep, and there were 4982 up-regulated and 4249 down-regulated circRNAs (Figure 3B, Table S3). To validate the accuracy of differentially expressed circRNAs by RNA-seq data analysis, we randomly selected 10 circRNAs for RT-qPCR analysis. By analyzing the results, we found that the results of the two methods showed a high degree of consistency (Figure 3C). These results indicate that the differentially expressed circRNAs, we analyzed actually occurred in vivo. In order to determine the clustering pattern of differential expression circRNAs between the estrus state and anestrus state of sheep pituitary glands, we used the TPM values of the differentially expressed circRNAs in the samples from these two periods for hierarchical cluster analysis. We found significant differences in the expression patterns of circRNAs in the pituitary glands of the sheep during these two periods (Figure 3D).

### 3.5. Enrichment Analysis of Circular RNAs

**Figure 4:**
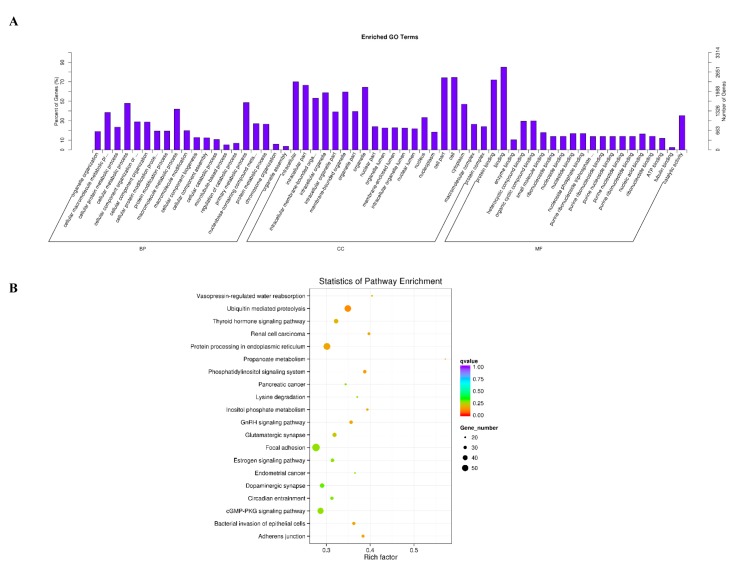
Enrichment analysis of circRNAs. (**A**) Gene ontology (GO) enrichment analysis of differentially expressed circRNAs (*p* < 0.05). The abscissa is the GO macromolecule and GO terminology at the next level, and the ordinate is the number and proportion of genes annotated to the term. (**B**) Differentially expressed host genes of circRNA were subjected to Kyoto Encyclopedia of Genes and Genomes (KEGG) enrichment analysis (*p* < 0.05). The vertical and horizontal axes represent the access name and access factor, respectively. The size of the dots represents the number of genes enriched in the visit, and the colors correspond to different q values.

The role of these differentially expressed circRNAs in the regulation of estrus and anestrus in sheep is unknown. To gain a deeper understanding of the role of this circRNAs, we used GO analysis to study the biological function of this circRNAs at an overall level. We found that a total of 956 GO terms were significantly enriched in the three categories of GO analysis (Table S4), including biological processes, cellular components and molecular functions. Interestingly, by further analysis we found that some GO terms that are closely related to gene expression and regulation are significantly enriched (Figure 4A), including primary metabolic process, cellular metabolic process, macromolecule metabolic process, cell, cell part, intracellular, binding, protein binding, catalytic activity and so on. Next, we further analyzed the signaling pathways involved in these differentially expressed circRNAs using KEGG enrichment analysis, and we hoped to get more information about the biological functions of these circRNAs in regulating sheep’s estrus. These differentially expressed circRNAs were significantly enriched in 38 signaling pathways (*p* < 0.05) (Table S5). By further analyzing the top 20 signaling pathways that are significantly enriched, we can see clearly that the estrus-related signaling pathways are significantly enriched, including circadian entrainment, estrogen signaling pathway, GnRH signaling pathway and thyroid hormone signaling pathway (Figure 4B). Interestingly, some related signaling pathways involving protein synthesis, secretion, transduction and regulation are also enriched, such as ubiquitin-mediated proteolysis, protein processing in the endoplasmic reticulum, propanoate metabolism, cGMP−PKG signaling pathway, inositol phosphate metabolism, lysine degradation and the phosphatidylinositol signaling system. At the same time, some signaling pathways related to neuromodulation are also enriched, including the dopaminergic synapse and the glutamatergic synapse.

### 3.6. Bioinformatics Analysis of CircRNA-miRNA Networks

**Figure 5:**
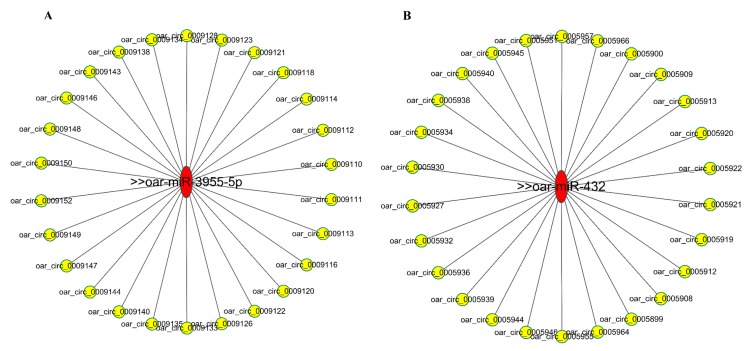
Predicting the interaction between circRNA-miRNAs. (**A**) Predicted circRNAs that interact with oar-miR-3955-5p. (**B**) Predicted circRNAs that interact with oar-miR-432.

To further understand the function of circRNAs involved in the pituitary regulatory network of sheep, the differentially expressed circRNAs identified in this study were predicted for miRNA-circRNA binding using the miRanda software. A total of 660,397 miRNA-circRNA potentially bindings were predicted (Table S6). It is worth noting that several well-known miRNAs are closely related to sheep estrus, which is considered to be the focus of future research, such as oar-miR-431 and oar-miR-3955. According to this study, we found that many of these differentially expressed circRNAs interact with miRNAs that regulate estrus. The potential miRNA-circRNA regulatory networks of oar-miR-431 and oar-miR-3955-5p were mapped by cytoscape 3.5.1 and showed a general correlation between microRNA and circRNA (Figure 5A,B). These predicted circRNAs are the focus of further research.

## 4. Discussion

The pituitary gland is a powerful and important endocrine organ that regulates mammalian estrus and reproduction primarily through the synthesis and secretion of hormones [28,29]. However, it has been reported that gonadal hormones and other hormones secreted by the pituitary gland are subjected to various levels of synthesis and secretion regulation [30]. In this study, circRNAs in pituitary glands involved in the regulation of sheep estrus were studied by high-throughput techniques (RNA-Seq). We identified a total of 12,468 circRNAs by bioinformatics analysis. Interestingly, we found a total of 9231 differentially expressed circRNAs in PGN and PGA. This shows a large number of difference in the expression pattern of circRNAs in the pituitary glands of sheep during estrus and anestrus, suggesting that circRNA may be widely involved in the regulation of these two states [30,31]. Furthermore, we demonstrated the presence and differential expression patterns of these circRNAs by RT-PCR and RT-qPCR, respectively. In addition, several randomly selected circRNAs treated with RNase-R in this study demonstrated the resistance of circRNA to RNase-R^+^ digestion due to their circular structure [32,33]. It is speculated that this particular structure confers stability to circRNAs, allowing them to function for long periods of time [34].

GO analysis indicated that most differentially expressed circRNAs involved protein processing, nuclear components and nucleic acid metabolism. At the same time, KEGG pathway analysis also revealed that circRNA participates in signaling pathways directly related to pituitary gland functions, including circadian entrainment [35], the estrogen signaling pathway [36], GnRH signaling pathway [37] and the thyroid hormone signaling pathway [38]. Interestingly, some related signaling pathways involving protein synthesis, secretion, transduction and regulation are also enriched, such as ubiquitin mediated proteolysis, protein processing in the endoplasmic reticulum, propanoate metabolism, the cGMP-PKG signaling pathway, inositol phosphate metabolism, lysine degradation and the phosphatidylinositol signaling system [39,40]. At the same time, some signaling pathways related to neuromodulation are also enriched, including the dopaminergic synapse and the glutamatergic synapse [36]. These conclusions indicate that the regulation of estrus in sheep is a process involving comprehensive regulation of multiple biological pathways, and more attention needs to be paid to this in the future.

Recent studies have shown that circRNA can function as a miRNA sponge or a potent competitive endogenous RNA (ceRNA) molecule in a range of biological processes [27]. The negative correlation between circRNA and miRNA is manifested by down-regulation of miRNA and up-regulation of circRNA or vice versa. Through this relationship, the function of the circRNA sponge is further confirmed by miRNA [32,41]. In addition, 660,397 circRNA-miRNA pairs were predicted and a large and complex network of interactions could be formed, although their working mechanisms remain unclear. At the same time, well-known oar-miR-431 and oar-miR-3955, which are closely related to sheep estrus, interact with many differentially expressed circRNAs. However, whether the above negative relationship exists between circRNA and miRNA in this study is still unknown. Overall, these differentially expressed circRNAs may regulate the function of the pituitary between estrus and anestrus by regulating miRNA, although specific mechanisms need further study in the future [42,43].

Of course, the large differences between the estrus and anestrus pituitary glands are also attributed to many other factors, such as proteins, intermediate metabolites and unknown small molecules [44,45]. However, this study does provide a new perspective for sheep breeding research and suggests new issues that require further research. At the same time, our research provides valuable insight into the biology of circRNA and helps to understand the function of circRNA in regulating physiological changes in animals.

## Data Availability

The datasets generated during and/or analyzed during the current study are available from the corresponding author on reasonable request.

## Supplementary Materials

The following are available online at www.mdpi.com/xxx/s1. Figure S1: Sequencing error rate distribution map, Table S1: The information of the primers used in this experiment, Table S2: Tshe information of circRNAs, Table S3: The differentially expressed circRNAs, Table S4: The differentially expressed circRNAs were analyzed by GO analysis, Table S5: KEGG pathway analysis demonstrated 266 terms were enriched, Table S6: All circRNA-miRNA interaction network information.
